# The journey to UHC: how well are vertical programmes integrated in the health benefits package? A scoping review

**DOI:** 10.1136/bmjgh-2021-005842

**Published:** 2021-08-03

**Authors:** Lydia Regan, David Wilson, Kalipso Chalkidou, Y-Ling Chi

**Affiliations:** 1 Global Health, Center for Global Development, London, UK; 2 Decision Sciences, Bill & Melinda Gates Foundation, Seattle, Washington, USA; 3 The Global Fund to Fight AIDS, Tuberculosis and Malaria, Grand-Saconnex, Switzerland

**Keywords:** health systems, HIV, immunisation, maternal health, malaria

## Abstract

**Background:**

Countries are recommended to progressively work towards universal health coverage (UHC), and to make explicit choices regarding the expansion of priority services. However, there is little guidance on how to manage the inclusion of vertical programmes, funded by external partners, in health benefits packages (HBP) in low and middle-income countries (LMICs).

**Objective:**

We conducted a scoping review to map the inclusion of six vertical programmes (HIV, tuberculosis, malaria, maternal and child health, contraceptives, immunisation) in 26 LMICs.

**Methods:**

We identified 26 LMICs with an HBP that was not aspirational (eg, with evidence of implementation or funding). For each HBP, we collected information on the corresponding UHC scheme, health financing at the time of establishment, revisions since inception and entitlements. For each vertical programme, we developed a list of tracer interventions based on the Disease Control Priorities 3 and the 100 Core Health Indicators List. We then used this list of tracer interventions to map the coverage of the six vertical programmes.

**Results:**

The review shows that there is no common starting point for countries embarking into UHC. Some HBPs were almost three decades old. Whole package revisions are rare. The inclusion of vertical programme does not follow a given pattern based on health financing indicators or country’s income group. Maternal child health services are the most often included and family planning the least. Six countries in our sample covered all vertical programmes, while one covered only one of six.

**Conclusions:**

This review has shown that there has been a long history of countries facing this question and we have provided the first mapping of inclusion of vertical programmes in UHC. The results of the mapping can inform decisions in countries embarking in UHC.

**Key questions**

**What is already known?**

- Countries are recommended to progressively work towards universal health coverage (UHC), and to make explicit choices regarding the expansion of priority services.
- As countries grow economically, donors reduce allocations or introduce changes in support modalities.
- There has been a global push to shift ownership to countries for developing, financing and delivering externally funded vertical programmes; the development of a UHC plan (and its accompanying health benefits package (HBP)) seems to be a good opportunity to achieve this.

**What are the new findings?**

- We have provided the first systematic mapping of inclusion of vertical programmes in UHC.
- The review shows that there is no common starting point for countries embarking into UHC, with HBPs almost three decades old to new pilots.
- There is not an observable pattern of inclusion based on countries’ health financing indicators, levels of development assistance or country’s income group.
- Maternal and child health services are most often included, and family planning least often included.
- Six countries in our sample covered all six vertical programmes.
- One country covered only one of six.

**What do the new findings imply?**

- This review has shown that there has been a long history of countries facing inclusion of vertical programmes within HBP, with no ‘common’ starting point regarding health system ‘maturity’, income group and levels of external support.
- This review shows that revisions are rare, and so vertical programme inclusion or exclusion within HBPs should be carefully considered at the outset of HBP design.

## Background

Historically, donors and multilateral organisations have channelled funding in health through vertical disease programmes, focused on one disease area with short-term and medium-term objectives. Vertical programmes have contributed to unprecedented reductions in mortality; in 2000 and 2015, new HIV infections fell by more than one-third^1^ and scaling up access to tuberculosis (TB) diagnosis and treatment between 2000 and 2017 averted an estimated total of 54 million deaths.^2^ While they present many advantages (eg, ease of management, greater accountability, strong financial control^3^), they have also been subject of controversy for over two decades. Criticisms included (1) the creation of parallel systems for funding and management, (2) a distortion of national priorities,^4^ (3) costly delivery,^3^ and (4) lack of contribution to strengthening of the healthcare system^5^; resulting to missed opportunities to build stronger health systems.^6^ As such, they have been described as ‘islands of sufficiency in a swamp of insufficiency’ by Ooms *et al*^7^ (2008).

For those reasons, there has been a global push to shift responsibility to countries for developing, financing and delivering externally funded vertical programmes. As countries grow economically, donors reduce allocations or introduce changes in support modalities (eg, increases in cofinancing) or policies and timelines to taper off funding—known as ‘transition from aid’.^8^ The contribution of development assistance for health (DAH) drops significantly as countries move from the status of low-income country (25.4% of total health expenditure (THE)) to lower middle income (3.2% of THE).^9^ Moreover, the economic shocks from COVID-19 in many high-income countries are likely going to translate into declines in DAH. This will have important consequences in countries or for programmes heavily relying on DAH: for example, previously close to 50% of the HIV/AIDS funding in low and middle-income countries (LMICs) came from DAH.^10^

Many countries have successfully managed their transitions while maintaining an overall trend of progress against the main disease burdens. However, the current cohort of nine countries projected to transition in the coming years is more unequal, poorer, more indebted and has weaker health systems than the previous cohort that transitioned between 2010 and 2015.^11^ Those concerns are likely to be exacerbated by the COVID-19 pandemic: the World Bank projects the largest contraction in global gross domestic product in decades (5.2%).^12^ Additionally, the rise of non-communicable diseases (NCDs) in transitioning LMICs has become a national priority requiring considerable resource needs, which are met through domestic rather than external funds (only 1%–2% of DAH goes to NCDs).^13^

Against this backdrop, many LMICs have committed to achieving the Sustainable Development Goals, including the target 3.8 of achieving universal health coverage (UHC). UHC is particularly high on the policy agenda of many transitioning countries, such as Kenya^14^ and Cote D’Ivoire.^15^ UHC includes a full spectrum of ‘essential, quality health services, from health promotion to prevention, treatment, rehabilitation and palliative care’.^16^ Central to UHC policy is the development of a health benefits package (HBP), a list of priority/essential services to be delivered through the wider health system, ideally chosen based on consistent and transparent criteria that reflect the UHC policy goals.^16^ The HBP creates sustainable entitlements to the covered population.

The development of a UHC policy could represent an opportunity to achieve the shift of ownership away from donors, through the *inclusion* of vertical programme services in the HBP design. *Inclusion* of vertical programmes is more extensive than *vertical integration* which concerns health service delivery across levels of care. It is also more expansive than *integrated service delivery*, which focuses on providing a full range of health services in the same location. The inclusion of vertical programmes would mean that those seeking care have one set of entitlements regardless of funding (domestic or external), integrated and managed in a single scheme. An additional potential advantage is that by integrating entitlements and therefore the system, with the view of integrating inputs (eg, procurement, infrastructure, staff, funding), it will improve service delivery, effective service coverage and sustainability.

In the last three decades, many countries with a significant reliance on external funding have developed UHC policies and HBPs. In Indonesia, the UHC scheme, the Jaminan Kesehatan Nasional (JKN), was launched in 2014, at a time when 60% of the total spending for HIV programmes came from external resources.^17^ In Rwanda, Community-Based Health Insurance covers more than three-quarters of the population and more than 50% of THE comes from external resources.^18^ A handful of studies have investigated the inclusion of specific vertical programmes in an HBP but with a single country focus and typically aiming to produce estimates of budget or fiscal impact (see Lee *et al*^19^). There has been no systematic study to assess whether and how countries included vertical programmes in the HBPs.^20^

This work aims to fill this gap by reviewing the inclusion of six vertical programmes in HBPs through a review of the literature. It is worth noting that we are focusing this review on the entitlements listed under the HBPs, and we are not able to comment on whether it translates into actual access of services for patients or whether the services are covered through another mechanism. We identified 26 countries that currently have an HBP at least partially implemented and resourced. (We exclude aspirational HBPs from this study. The selection process is further explained in the Methods section.) First, we present health financing indicators at the time of implementation to understand differences in countries embarking in UHC. Second, we collect information on the shape of the HBP. Finally, we map the coverage of six specific vertical programmes (HIV, TB, malaria, maternal and child health (MCH), contraceptives and immunisation) in the HBP.

## Methods

### HBP identification

We conducted a literature search using PubMed, Cochrane Library, Web of Science databases and Google between May and November 2020 using the following keywords: ‘Basic benefit package’, ‘Health benefit package’, ‘Basic Health Service Package’, ‘Essential health care package’, ‘Universal health insurance’. We identified 30 countries in this initial search. In addition, we relied on existing HBP reviews and guidance (including Glassman *et al*^16^ and the US Agency for International Development’s (USAID) Health Finance and Governance (HFG) series on Essential Package of Health Services Country Snapshots) to identify HBPs not documented in the published literature. Nine countries were added through this additional step. In total, the search identified a total of 39 HBPs in LMICs. Data were extracted independently by two of the authors, although not in duplicate.

We excluded HBPs based on the two following criteria: (1) country income group (only included LMICs at the time of establishment) and (2) aspirational HBPs. To this end, we used the World Bank groupings^20^ with the year of HBP development to exclude high-income countries.

The exclusion of aspirational HBPs was more difficult, as many LMICs have developed an essential package of care list and sources of funding for those are not always clearly presented. We searched the literature on UHC to find appropriate rules for distinguishing aspirational and implemented HBPs but did not find any. As a result, we developed our own following rule: we collected the following information: (1) pooled funding to support the delivery of the HBP, (2) funding lines to reimburse or pay decentralised authorities or healthcare providers for the delivery of the HBP, and (3) legislation or regulation on the UHC policy or HBP entitlements. If evidence for at least one of the above variables was not found, the HBP was considered aspirational. This led to the exclusion of 13 countries and our final sample consists of 26 HBPs. Where several HBPs were found to fit the selection criteria for a given country, we selected those which (1) covered the largest share of the population or (2) central-level packages as opposed to state or regional ones. This is particularly relevant in countries with very large populations and regional or state-level packages, such as India and China. For example, India’s Pradhan Mantri Jan Arogya Yojana (PMJAY) package does not include immunisation, therefore it was considered not included even if some regional benefit packages do include immunisation.

### Information sources

For every HBP identified through the search, we collected the information in table 1.

**Table 1:** Information collected

	Data collected	Source
Retrospective landscape analysis	Year of establishment (abbreviated year of establishment)	Ministry of Health reports, government legislation, insurance fund administrative documents
	Description of HBP entitlements	Government published documentation
	Income group at year of establishment	World Bank classifications
	Government spending on health as % of THE at year of establishment	WHO DataBank
	Out-of-pocket spending as % of THE at year of establishment	WHO DataBank
	External spending on health as % of THE at year of establishment	WHO DataBank
	Sources of financing (when available) at year of establishment	Donor reporting documentation, evaluations of budget support Government published documentation, country mid-term expenditure reports
HBP revisions	HBP revisions: year	Government published documentation, peer-reviewed papers, peer-reviewed literature or grey literature (country reviews and case studies)
	HBP revisions: nature of the revisions	Government legislation, Ministry of Health documentation, peer-reviewed papers, grey literature (country reviews and case studies)
HBP coverage of vertical programmes	Coverage of HIV/AIDS interventions	Government published documentation, peer-reviewed papers, grey literature (country reviews and case studies)
	Coverage of malaria interventions	Government published documentation, peer-reviewed papers, grey literature (country reviews and case studies)
	Coverage of TB interventions	Government published documentation, peer-reviewed papers, grey literature (country reviews and case studies)
	Coverage of MCH interventions	Government published documentation, peer-reviewed papers, grey literature (country reviews and case studies)
	Coverage of contraceptives	Government published documentation, peer-reviewed papers, grey literature (country reviews and case studies)
	Coverage of immunisation	Government published documentation, peer-reviewed papers, grey literature (country reviews and case studies)

‘Government published documentation’ includes Ministry of Health or Health Insurance Fund legislative documents, website pages, reports, decrees, administrative documents or press releases.

HBP, health benefits package; MCH, maternal and child health; TB, tuberculosis; THE, total health expenditure.

For health financing information under the category *retrospective landscape analysis*, we used the World Bank’s World Development Indicators. For all other information, peer-reviewed papers and reports including Ministry of Health literature using the names of benefit packages and countries of interest and global public good literatures (eg, HFG’s country snapshots series) were used to collate information on the source of financing, revisions, content of the HBP and the coverage of the six vertical programmes. All data used in this paper are public and a list of reference can be supplied on request to the authors.

### Classification of inclusions

This review covers six vertical programmes: HIV, malaria, TB, MCH, immunisation and contraception. *Contraception* was separated from *MCH* due to the distribution of contraceptives often being financed and managed separately. Similarly, *immunisation* was separated because of the historic precedent of vertical vaccination campaigns.

To map the coverage of each vertical programme, we defined a set of tracer interventions that are (1) widely considered key components of the wider package and, (2) when applicable, cover services across the continuum of care for the considered package. The use of tracer interventions was necessary given the high number of interventions in each of the six packages, which would have made it difficult to capture consistently across our sample of 26 countries. It allows us to focus on a manageable set of interventions without reviewing the entirety of the components in each package.

We anchored this definition of tracer interventions in the Disease Control Priorities 3 (DCP3) Essential UHC (EUHC).^21^ EUHC is a subset of 213 interventions that have been identified as the highest priority based on a comprehensive synthesis of epidemiological and economic evidence and expert opinion. EUHC interventions were selected based on value for money (not exclusively cost-effectiveness), priority given to the worse off and financial risk protection. Of those 213 interventions, 53 fell within the scope of the six vertical programmes. We further prioritised and refined the list through crosswalks with the 100 Core Health Indicators List,^21^ literature reviews, expert opinion and piloting of the list in five countries in our sample. Our final list contains 14 services presented in online supplemental file 1. For instance, we selected the indicator ‘Detection and treatment of childhood infections (iCCM), including of referral if danger signs’ as a tracer intervention because three-fourths of deaths for under five are due to common infections and that interventions to tackle those infections can be delivered effectively and safely through community care.^22^

10.1136/bmjgh-2021-005842.supp1 Supplementary data

In some countries, entitlements were described as partially explicit (see table 2). In those countries, additional research had to be carried out by researching the tracer indicators and the name of the scheme. One example is Thailand, where the Universal Coverage Scheme covers all primary care, except for services on a negative list. As an example, in this case, we researched the keywords ‘contraceptives’ AND ’Universal Coverage Scheme Thailand’ to document the inclusion. Where possible, we requested country technical staff identified through contacts to validate the collected information.

**Table 2:** Shape of HBP packages

Shape	Description	Countries
Explicit	A well-defined list of services	Afghanistan, Argentina, Bangladesh, Colombia, Dominican Republic, Ethiopia, Ghana, Kenya, Mexico, Philippines, Rwanda, Uganda, Uruguay, Zambia
Partially explicit	Covering disease areas with little information on what is covered and what is not	China, Peru, Chile, India, Indonesia, Morocco, Thailand, Vietnam, Lebanon, Senegal
Implicit	Very broad entitlement, defined by access to type of health facility, or ‘all prevention and promotion services’ with no further specificity	Honduras, Kazakhstan

HBP, health benefits package.

Once the content was recorded, we developed a traffic light system to describe the inclusion and exclusion of the vertical programmes. The modalities of inclusion are described in table 3.

**Table 3:** Traffic light system: classification of inclusions

	Highest form of inclusion: all tracer interventions are covered.
	Medium form of inclusion: the majority (but not all) of tracer interventions are covered.
	Low form of inclusion/no exclusion: only one or no tracer intervention was covered in the package.
	Information unavailable or not applicable.

## Results

In this review, we identified 26 countries where evidence of implementation of an HBP was found. Nine were in Asia, seven in South America, nine in Africa and one in the Caribbean. In the time between establishment of the HBP and the current day, 11 countries transitioned to higher income status: 4 countries transitioned from low to lower middle income, 5 from lower middle to upper middle and 2 from upper middle to high income.

Table 4 presents the basic information on the year of establishment, major revision and health financing information at the time of implementation.

**Table 4:** Health financing data

Country	Name	Year established	Major revision to entitlements	Income group at year of establishment	Government spend on health at year of establishment (% THE)	External spend on health at year of establishment (% THE)
Afghanistan	Basic Package of Health Services (previously *Essential Package of Hospital Services* and *Integrated Package of Health Services*)	2003	2019	Low	7.30	6.60
Argentina	Argentina Plan Nacer/SUMAR	2004	2015	Upper middle	52	6.30
Bangladesh	Essential Package of Health Services (EPHS) (previously *Essential Service Delivery Package* (*ESD*), *Health, Population and Nutrition Sector Development Programme* (*HPNSDP*))	1998		Low	28.70	8
Chile Acceso Universal a Garantías Explícitas/Garantías Explícitas en Salud (AUGE/GES)	2005	2006, 2007, 2010, 2013	Upper middle	39	0.20	
China	New Rural Co-operative Medical Scheme	2002		Lower middle	25.40	0.15
Colombia	Mandatory Health Plan (POS)	1993	2012, 2015	Lower middle	75	0
Dominican Republic	Health Services Plan (PDSS) (previously *Basic Health Plan*)	2001	2016	Lower middle	61	3
Ethiopia	Essential Health Services Package (EHSP), building on Health Service Extension Program	2005	2019	Low	42	22
Ghana	National Health Insurance	2003		Low	29	13
Honduras	Honduras Basic Health Package (PBS)	2003		Lower middle	37.90	9.50
India	Pradhan Mantri Jan Arogya Yojana (PMJAY) (previously *Rashtriya Swasthya Bima Yojana*)	2008	2018	Lower middle	20.90	1.50
Indonesia	Jaminan Kesehatan Nasional (JKN)	2014		Lower middle	34.50	2.00
Kazakhstan	State Guaranteed Health Benefits Package (SGHBP)	2009	2014	Upper middle	76	0.30
Kenya	Universal health coverage (pilot, under the National Hospital Insurance Fund)	2018		Lower middle	42.70	17.90
Lebanon	Essential healthcare benefit package (EHCP)	2016		Upper middle	49.70	0.80
Mexico	Mexico’s Seguro Popular	2003	2013–2018	Upper middle	41.50	0.0*
Morocco	Régime d’Assistance Médicale (RAMED)	2012		Lower middle	42.50	0.60
Peru	The Essential Health Insurance Plan (PEAS)	2009	2012	Upper middle	50	2.10
Philippines	National Health Insurance Program (NHIP), PhilHealth	1995	2000, 2003, 2014	Lower middle	44	3.50
Rwanda	Mutuelles de Santé/Community-Based Health Insurance (CBHI)	2004	2011	Low	25.70	47
Senegal	Couverture Maladie Universelle	2013		Lower middle	26.70	9.40
Thailand	Thailand universal healthcare	2002		Lower middle	62	0.20
Uganda	Uganda National Minimum Health Care Package (UNMHCP)	1994		Low	24.70	27.40
Uruguay	The Comprehensive Health Care Plan (PIAS)	2008		Upper middle	49	0.06
Vietnam	Basic Package of Health Services (BPHS)	2009		Lower middle	36.2	2.5
Zambia	National Health Insurance Scheme	2020		Lower middle	39.1†	44.6

For health benefits packages (HBPs) established prior to 2000, we used the closest available year.

*There were no available data from the WB Databank for external expenditure for Mexico. For this country instead we use the WHO Global Health Expenditure Database (GHED), but the definitions are different between the two sources (GHED using current health expenditure and WB Databank using THE).

†Data from 2018 were used rather than 2020 as it was the closest available year.

THE, total health expenditure.

The oldest HBP found was the Mandatory Health Plan in Colombia (founded in 1993) and the latest one in Zambia (2020). There is no clear ‘starting point’ for countries embarking in UHC. Half of the countries were lower middle-income countries at year of establishment, and respectively seven (27%) and six (23%) were upper middle income and low income. Government expenditure on health (as % THE) varied between 7.3% in Afghanistan and 76% in Kazakhstan. Similarly, external spend (as % THE) ranged from 0% in Colombia to 47.0% in Rwanda, and this figure was higher in countries in the sub-Saharan region.

Out of 26, only 12 HBPs were subject to a major revision to their entitlements since their inception. (Other HBPs may have had other revisions, for example, increasing coverage population of the HBPs.) For instance, Ghana and Honduras have both established an HBP in 2003, which has not been subject to a major revision to date. On the other hand, Chile’s AUGE/GES has undergone a progressive expansion with increased entitlements almost every 3 years to match the increasing fiscal space for the programme.^23^ It is worth noting that from the onset, this programme had a written provision by law for reviewing the package every 2 years.^16^ Interestingly, we found that revisions were mostly additions to the existing package: only PMJAY in India seemed to have gone through a review that led to significant deprioritisation. In 2019, a total of 554 packages were discontinued from PMJAY.^24^

The review highlighted many shapes of HBP, in other words, different ways of defining of entitlements (described in table 2). The most frequent shape (14 countries, 50%) was explicit: meaning the HBP is made of a well-defined list of services such as ‘identification and management of obstetric complications (eg, haemorrhage or puerperal infection/sepsis)’ (as found in Bangladesh). Ten countries (38%) adopted partially explicit shapes, specifying disease areas but with little further detail on what was covered. Two countries had entitlements defined based on categories or levels of care.

Table 5 shows the traffic light system used to categorise the inclusions of the six vertical programmes in HBPs. It was not possible to document traffic lights for Kazakhstan and Honduras given the HBPs were completely implicit, and for China, where detailed English language publications for a centralised HBP as opposed to state-level HBP were not found.

**Table 5:** Traffic light inclusion of vertical programmes within country HBPs*

	HIV	Malaria	TB	MCH	Immunisation	Contraception
Afghanistan						
Argentina						
Bangladesh						
Chile						
China						
Colombia						
Dominican Republic						
Ethiopia						
Ghana						
Honduras						
India						
Indonesia						
Kazakhstan						
Kenya						
Lebanon						
Mexico						
Morocco						
Peru						
Philippines						
Rwanda						
Senegal						
Thailand						
Uganda						
Uruguay						
Vietnam						
Zambia						

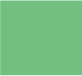
 All tracer interventions covered; 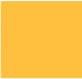
 most but not all tracer interventions covered; 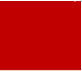
 one or no intervention covered; 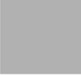
 not available or not applicable,

*Full database of references used for the mapping can be shared on request.

HBP, health benefits package; MCH, maternal and child health; TB, tuberculosis.

The inclusion of vertical programmes follows no discernible pattern. Six countries (23%) included fully all six vertical programmes and those six countries had initiated their HBP at very different stages: for instance, while the two countries cover all six programmes, Rwanda is a low-income country where 47.0% of THE came from external sources (at the time of establishment) and Mexico was an upper middle-income country where external spend did not significantly contribute towards THE. By grouping countries by levels of external funding at establishment (<1%, 1%–10% and >10%), we can see the levels of inclusion on average are similar. For packages with external funding at the time of HBP establishment between 1% and 10% and those above 10%, the average for number of full inclusions (ie, green in table 5) was at 3.8. For countries with external aid less than 1%, and had lower levels of inclusions, the average was 3.3 green inclusions. Uruguay had the lowest coverage for those six packages, including only immunisation. Ghana was another country where inclusion of the six packages was more limited: with coverage of some MCH services and full coverage of malaria.

In terms of programmes, MCH was the most included (categorised as green in 19 countries), followed by HIV (green in 16 countries) and malaria (green in 14 countries). Contraception was the least included (green in 11 countries). It is worth noting immunisation was least often ‘fully’ included (classified as orange in five countries). Only one intervention was covered in all packages where information was available: ‘Management of labour and delivery by skilled attendants, including obstetric delivery, delivery complications, basic neonatal resuscitation’ (see online supplemental file 1 for the full list of interventions).

## Discussion

COVID-19 has highlighted the critical importance of countries building resilient health systems towards achieving UHC.^25^ As part of building UHC, developing an HBP that secures the maximum value (whether greater health, equity or any consideration), while remaining within the available funding, is a critical factor for the policy’s success.^16^ In this piece, we review how countries have prioritised inclusions of vertical programmes in HBPs, a question of much interest as many countries that receive substantial funding from donors are now embarking in UHC. This is, to our knowledge, the first review of addressing this question.

First, our review shows that there is a long history of HBP development shown in the 27-year range of HBP establishment. We also found that there is no ‘common’ starting point at which HBPs are developed: different countries at different stages of ‘maturity’ of their health system (as proxied by the % of THE from government sources), income status (low, lower middle, upper middle) and external support have developed HBPs that were more than aspirational. Despite many HBPs being over 20 years old, only 12 of the HBPs examined had well-documented revisions or adjustments to their entitlements. Uganda, for example, has not revised the Uganda National Minimum Health Care Package well in over two decades.^26^ This was not an uncommon situation and it emphasises the need to formally consider actively the inclusion of vertical programmes ahead of transition or build in a revision process that ensures their consideration as the country transitions and health needs evolve.

We found different patterns of inclusion for the six vertical programmes considered, and only six countries included all six programmes. That MCH was the most often included is unsurprising. There are high levels of global commitment to MCH; in 2019 33% of all DAH was for MCH, at a global total of $13.3 billion.^27^ Primary healthcare is recognised as the most cost-effective way to reach UHC,^28^ and many countries have committed to MCH interventions to reach this goal.^29^ By contrast, family planning (although in our review only the provision of a range of contraceptive commodities) was the least included. While family planning is often grouped with other services under the umbrella of reproductive, maternal, newborn, child and adolescent health, this analysis shows that its consideration within the HBP follows a very different pattern to MCH. This finding is consistent with a recent review on family planning inclusions in HBPs showing that most emerging health insurance schemes exclude contraception from reimbursable benefits packages.^30^

In many instances, we also found partial inclusions of the vertical programmes, even when only mapping interventions that were widely considered essential and cost-effective within each programme. This points to modalities of inclusion that are context specific. Understanding how different contextual factors shaped those decisions will be an important contribution of future research.

Finally, we could not find a correlation between DAH and inclusions: one initial hypothesis was that the countries that had included vertical programmes had already transitioned out of aid. For instance, Chile developed AUGE/GES at the time when DAH contributed less than 1% of THE but excluded the six programmes out of the HBP. Rwanda, one of the countries where the contribution of DAH (as % THE) is the highest in the world, has included all six programmes as part of its package. When comparing countries in three categories of external financing, we observed very similar levels of inclusion, with countries with less than 1% external aid at the time of establishment having only slightly less full inclusions in their HBP.

The decision to include entirely, partially or to exclude could be explained by many factors. First, as pointed by Glassman *et al*,^16^ the total size of the HBPs needs to match the available funds for the UHC policy. Adding more components of vertical programmes has important financial implications: in a recent survey of key stakeholders from the government in Ghana, respondents expressed concerns about the financial gap left by donors and consequently possible interruptions of care for the beneficiaries.^31^ They suggested that covering more through to the National Health Insurance Agency could be a solution, although recognising the existing concerns about the scheme’s financial sustainability as it had been running a deficit over several years.^32^ A recent review of JKN in Indonesia also highlights that while the integration of HIV and TB services into the HBP is ‘preferable’ (p 19), careful consideration of the costs, resource implications, feasibility (eg, given health system constraints) and sources of funding will need to inform what services are included.^17^

Other considerations may also influence the decision. On family planning, Marshall^33^ postulates that in some countries, religion plays a factor in government’s choices of implementing certain family planning policies and reforms above others. Family planning may also be more frequently excluded given the high levels of donor dependency (especially for financing commodities)^34^ and low cofinancing requirements.^35 36^ Finally, information required to prioritising a wide range of interventions may not have been as widely available for older HBPs. In a recent review of Ethiopia’s Essential Health Services Package, the prioritisation process started from consideration of 1749 interventions using the WHO intervention compendium and the DCP3.^37^ It could be that some of the earlier HBPs adopted a narrower focus for this reason.

Finally, the inclusion of certain programmes may also be influenced by the ability of donors to support the UHC policy overall. While it was not the focus of this review, we have identified different mechanisms that have been trialled by countries: coverage of copayment, sourcing of commodities, pooling through sector-wide approaches (SWAps). For instance, in Vietnam, the US President’s Emergency Plan for AIDS Relief and The Global Fund to Fight AIDS, Tuberculosis and Malaria have both funded copayments for HIV clients to the Social Health Insurance (SHI)^38^ and sourced commodities. As a result, the Government of Vietnam will now cover the antiretrovirals by 2021 through SHI.^39^ Budget support through pooled resources to support the UHC policy has occurred in Bangladesh and Afghanistan. In Bangladesh, donors contributed to SWAps to health since the development of Essential Package of Health Services starting at almost 50% contribution, which has declined to 34% in the current (third) SWAp.^40^ Similarly, in Afghanistan, the European Union, USAID and World Bank committed to providing long-term funding from the outset to the Basic Package of Health Services in 2002, although external contributions plateaued after 2008.^41^ It is worth noting in both cases, donors jointly agreed on priorities and inclusions with countries and perhaps as a result, both Bangladesh and Afghanistan had the broadest inclusions in this review. Dalil *et al*^41^ highlighted that part of the success of the experience of Afghanistan was the strong mutual accountability between the Ministries and donors. A mapping and evaluation of these different methods of support could inform a constructive dialogue between countries and agencies funding vertical programmes. Research into those modalities and potential innovations should be undertaken to further advance those conversations.

We note several limitations to this study. First, our review may have failed to identify HBPs due to the occasional unavailability of original documentation and that our literature search was conducted primarily in English although where possible, sources not published in English were translated using a document translation browser extension to give an idea of the content of documents. Moreover, we sought to separate aspirational HBPs from those implemented in practice but have found that the distinction was very difficult to apply consistently across countries. Instead, we used three variables (described in the Methods section) to select countries that had an HBP with ‘evidence of implementation’, but this methodology was not applied in other studies. However, we did not find any attempts to separate aspirational from implemented HBPs in the literature. Moreover, we fully recognise that listing an intervention under the HBP does not mean that the service is necessarily provided. We were only able to review the entitlements as defined in the HBP. Glassman *et al*^16^ also distinguish between such entitlements *de jure* from the *de facto* HBP that ‘patients actually receive’. Conversely, our review recognises that if a service is not explicitly listed in official documentation, it does not necessarily follow that the service is not provided through other government channels (ie, a separate programme). Our review of entitlements has a narrow focus, but it allows us to compare and contrast country experiences and vertical programmes and it will be helpful in informing conversations about definition of entitlements in HBPs. There may also be some inaccuracies for countries where the HBP was only partially implicit: targeted queries were carried out to inform the mapping when it was the case. If the information was not documented from the Ministry or in external literatures, then it was likely to not have been accurately captured in the study.

Finally, one of the original intents of this piece was to consider how HBPs had evolved from their establishment to the present date. This would have involved mapping the original HBP and additions across time. For example, Thailand gradually included HIV services into their HBP as the fiscal space allowed.^42^ However, analysis was not possible due to poor levels of documentation on the evolution of HBPs and the difficulty in tracing back documents sometimes over 20 years.

## Conclusions

Countries are recommended to progressively work towards UHC, and to make explicit choices regarding the expansion of priority services.^43^ However, there is little guidance on how to manage the inclusion of vertical programmes in the HBP. This review has shown that there has been a long history of countries facing this question and we have provided the first systematic mapping of inclusion of vertical programmes in UHC. One important result is that the inclusion of vertical programme does not necessarily follow a given pattern, although MCH services are most often included and family planning least often so. Moreover, our review shows that revisions are rare, and so vertical programme inclusion or exclusion within HBPs should be carefully considered from the outset of HBP design.

## Data Availability

All data relevant to the study are included in the article or uploaded as supplemental information. The database of references used for the mapping has been uploaded as supplemental information.
