# Supplementary material for: The journey to UHC: how well are vertical programmes integrated in the health benefits package? A scoping review
Source: BMJ Glob Health. 2021 Aug 3;6(8):e005842. doi: 10.1136/bmjgh-2021-005842 (PMC8336212; doi:10.1136/bmjgh-2021-005842)
Supplement: Supplementary data [file bmjgh-2021-005842supp001.pdf]

Annex 1. List of 14 tracer interventions

**HIV**	HIV testing and counselling (including in the community), with appropriate referral or linkage to care
	Among all individuals who are known to be HIV positive, immediate ART initiation with regular monitoring of viral load for adherence and development of resistance
	PMTCT of HIV (Option B+) and syphilis
**Malaria**	In malaria-endemic settings, diagnosis of malaria by rapid diagnostic test or microscopy
	Treatment with ACTs (or current first-line combination) or relevant oral/rectal anti-malarial medicines upon positive diagnosis
	In high malaria transmission settings, intermittent preventive treatment in pregnancy
	In malaria-endemic settings, provision of insecticide-treated nets to children and pregnant women attending health centres
**TB**	Diagnosis of TB, including assessment of rifampicin resistance
	Initiation of first-line treatment per current WHO guidelines for drug-susceptible TB; referral for confirmation, further assessment of drug resistance, and treatment of drug-resistant TB
**Maternal and Child health**	Detection and treatment of childhood infections (iCCM), including referral if danger signs. Full supportive care for severe childhood infections with danger signs
	Management of labour and delivery by skilled attendants, including obstetric delivery, delivery complications, basic neonatal resuscitation
	Early detection and treatment of neonatal complications and infections, including meningitis, pneumonia, sepsis
**Immunisation**	Childhood vaccination series (diphtheria, pertussis, tetanus, polio, BCG, measles, hepatitis B, Hib, rubella)
	Pneumococcus vaccination
**Contraception**	Provision of condoms and hormonal contraceptives, including emergency contraceptives

Regan L, *et al. BMJ Global Health* 2021; 6:e005842. doi: 10.1136/bmjgh-2021-005842
